# Supplementary material for: NIR light-activated nanocomposites combat biofilm formation and enhance antibacterial efficacy for improved wound healing
Source: Commun Chem. 2024 Jun 8;7:131. doi: 10.1038/s42004-024-01215-1 (PMC11162491; doi:10.1038/s42004-024-01215-1)
Supplement: Supplementary file 2 — Supplementary Information [file 42004_2024_1215_MOESM2_ESM.pdf]

Supplementary Information

NIR light-activated Nanocomposites Combat Biofilm Formation and Enhance Antibacterial Efficacy for Improved Wound Healing

Irfan Ullah[1,6], Shahin Shah Khan[1,6], Waqar Ahmad[1], Luo Liu[1], Ahmed Rady[2], Badr Aldahmash[2], Yingjie Yu*[3], Jian Wang*[4], and Yushu Wang*[5]

[1] College of Life Science and Technology, Beijing University of Chemical Technology, No. 15 East Road of North Third Ring Road, Chao Yang District, Beijing 100029, China

[2] Department of Zoology, College of Science, King Saud University, P.O. Box 2455, Riyadh 11451, Saudi Arabia

[3] State Key Laboratory of Organic-Inorganic Composites, Beijing University of Chemical Technology, No. 15 East Road of North Third Ring Road, Chao Yang District, Beijing 100029, China

Email: yuyingjie@mail.buct.edu.cn

[4] Department of Head and Neck Surgery, National Cancer Center/National Clinical Research Center for Cancer/Cancer Hospital, Chinese Academy of Medical Sciences and Peking Union Medical College, Beijing 100021, China

Email: wangjianpumc@126.com

[5] The People's Hospital of Gaozhou, National Drug Clinical Trial Institution, Gaozhou City 525200, China

Email: wysmjeda@gmail.com

[6] Both authors contributed equally to this work.

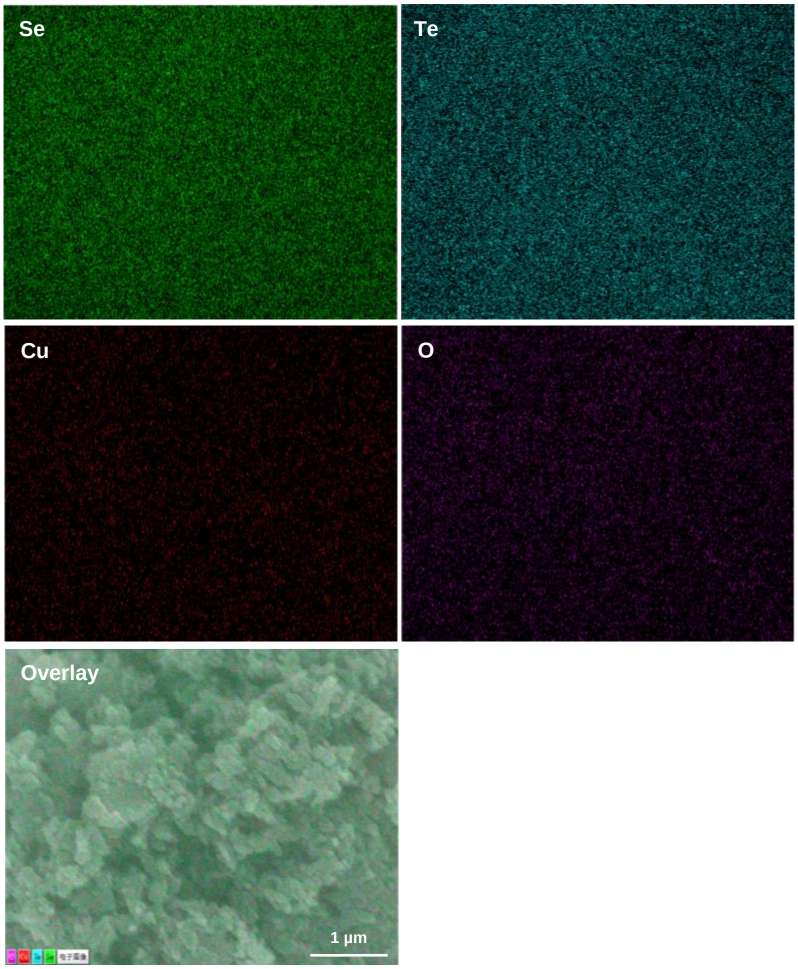

Supplementary Figure 1. EDS pictures of SeTe-CuO NPs.

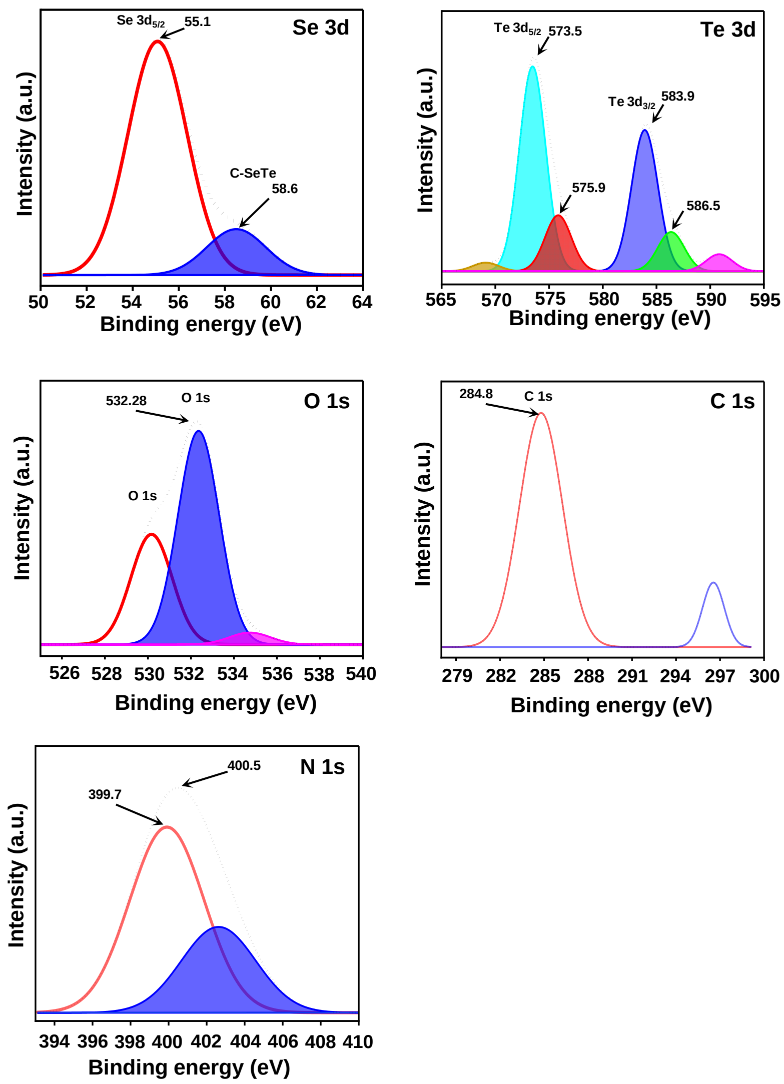

Supplementary Figure 2. XPS elemental spectra of SeTe-CuO NPs.

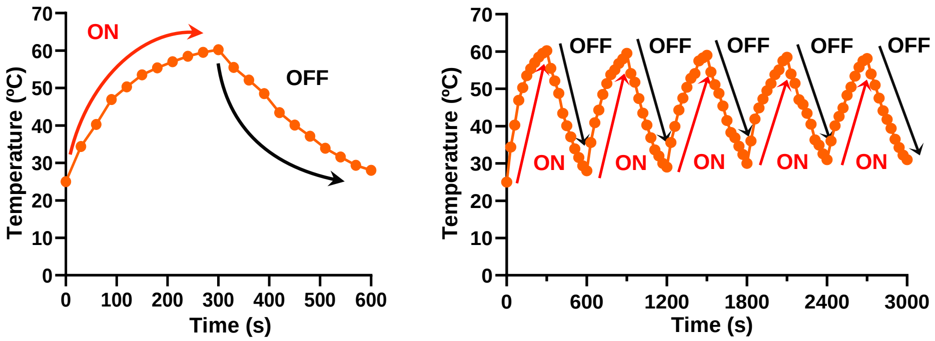

Supplementary Figure 3. Single and five consecutive cycles of SeTe-CuO NPs + L with 5 min NIR laser irradiation ON and then OFF.

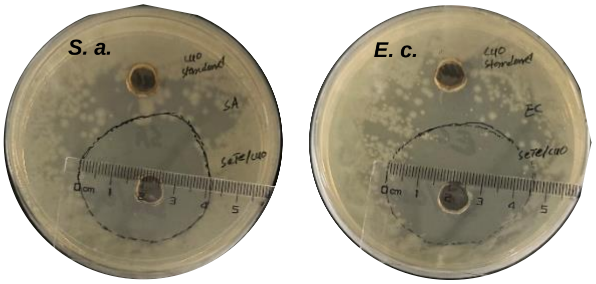

Supplementary Figure 4. Zone of inhibitions of SeTe-CuO NPs against *S. aureus* and *E. coli.*

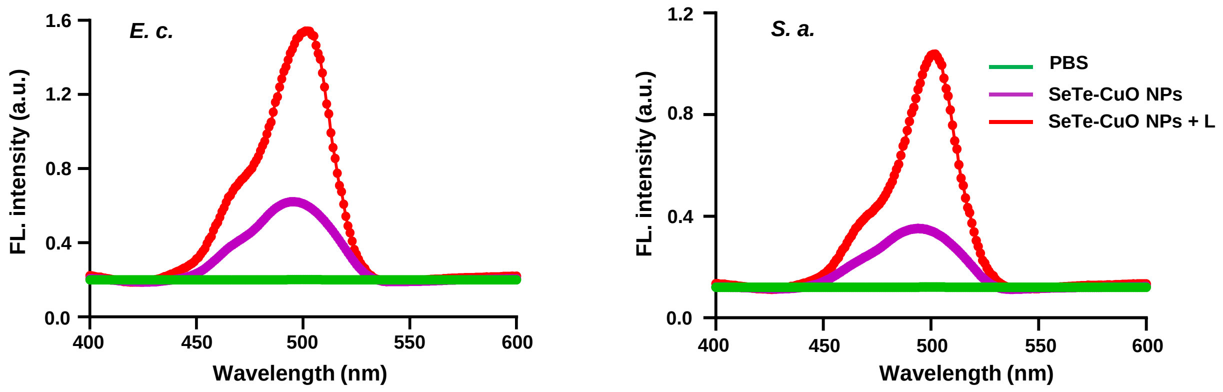

Supplementary Figure 5. Fluorescence intensities (FL) of intracellular ROS generation of *E. coli* and *S. aureus* after treatment with SeTe-CuO NPs and SeTe-CuO NPs + L.

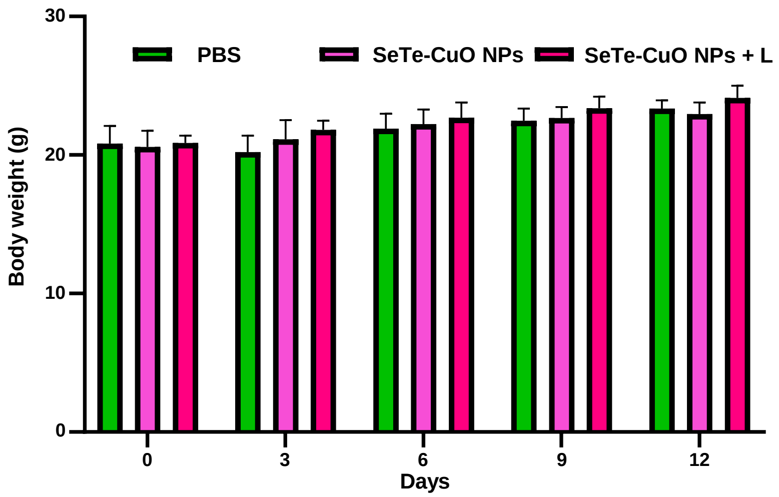

Supplementary Figure 6. Body weight of mice after treatment with PBS, SeTe-CuO NPs and SeTe-CuO NPs + L. The data represent mean values and the error bars correspond to standard deviations.

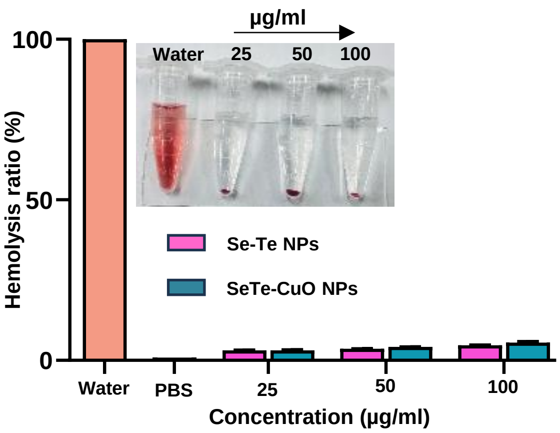

Supplementary Figure 7. Hemolysis ratio of the mice blood samples after treatment with Se-Te NPs and SeTe-CuO NPs. The data represent mean values and the error bars correspond to standard deviations.
