# Supplementary material for: NIR light-activated nanocomposites combat biofilm formation and enhance antibacterial efficacy for improved wound healing
Source: Commun Chem. 2024 Jun 8;7:131. doi: 10.1038/s42004-024-01215-1 (PMC11162491; doi:10.1038/s42004-024-01215-1)
Supplement: Supplementary file 3 — Description of Additional Supplementary Files [file 42004_2024_1215_MOESM3_ESM.pdf]

Description of Additional Supplementary Files

File name- Supplementary Data 1

File Description- All the numerical data used for making graphs in the main manuscript as well as in supplementary information.
